# Supplementary material for: The Escalating Effects of Wildlife Tourism on Human–Wildlife Conflict
Source: Animals (Basel). 2021 May 12;11(5):1378. doi: 10.3390/ani11051378 (PMC8150641; doi:10.3390/ani11051378)
Supplement: Supplementary file 1 [file animals-11-01378-s001.zip › animals-1210968-supplementary.pdf]

Supplementary Materials

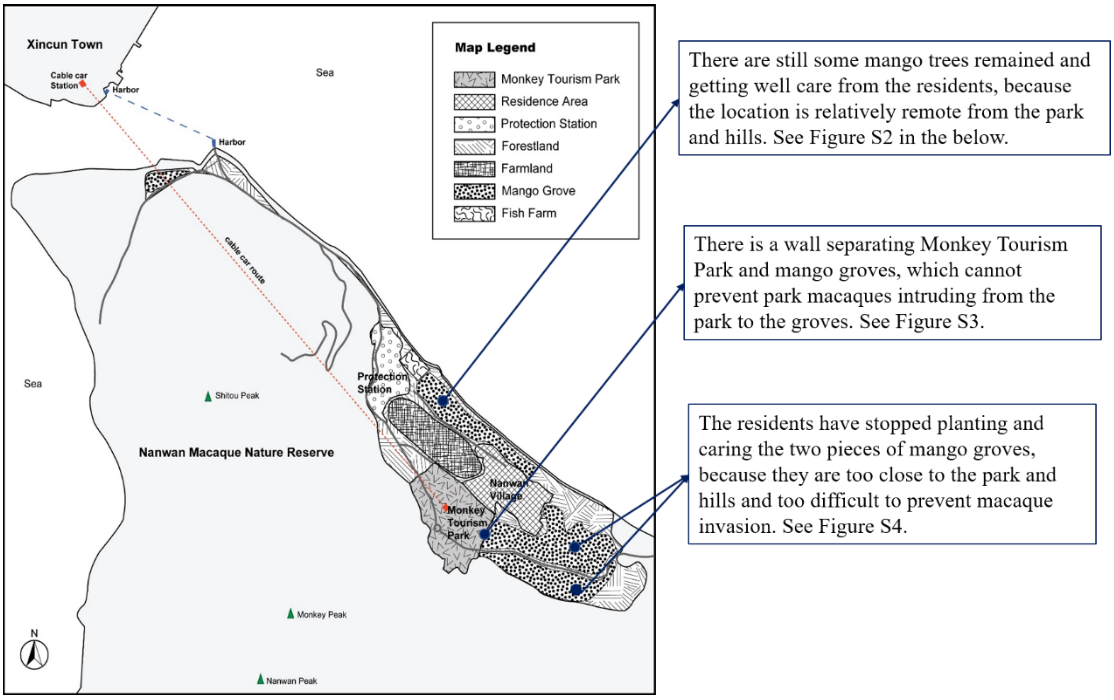

Figure S1. A photographic illustration of the community–macaque conflict

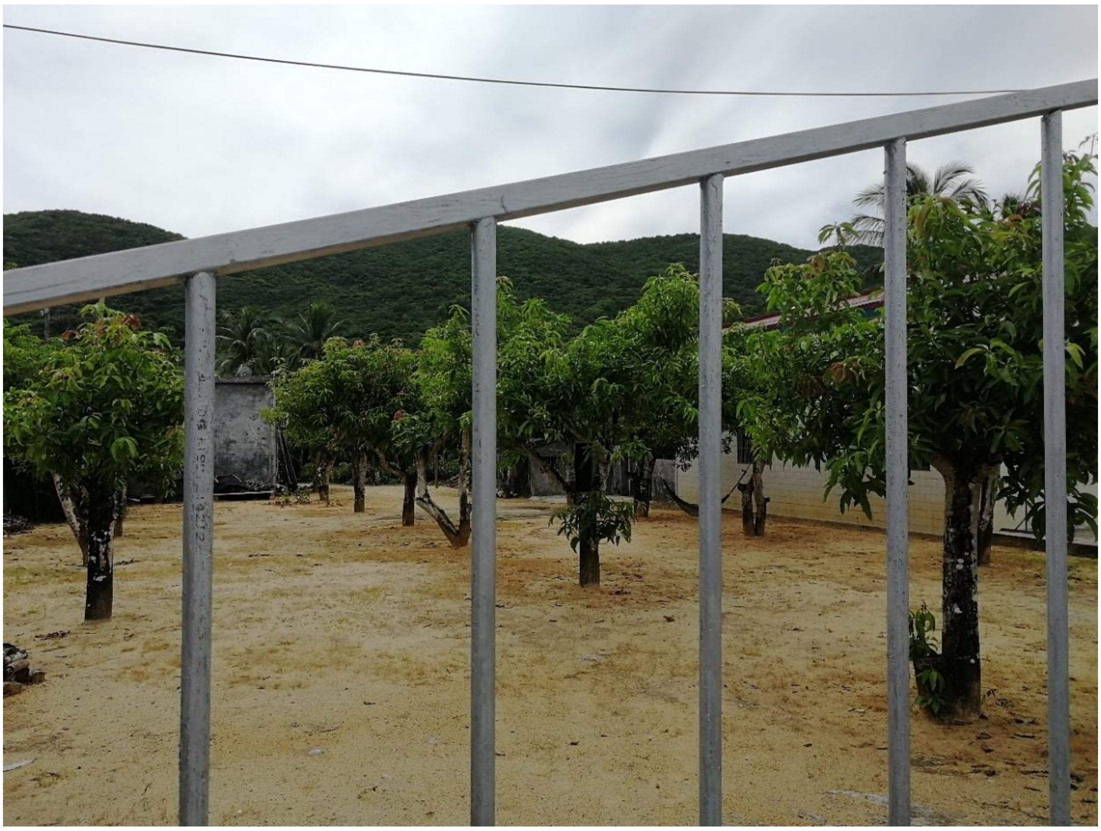

Figure S2. Well-cared mango groves in a relatively remote place from the monkey park and reserve hills

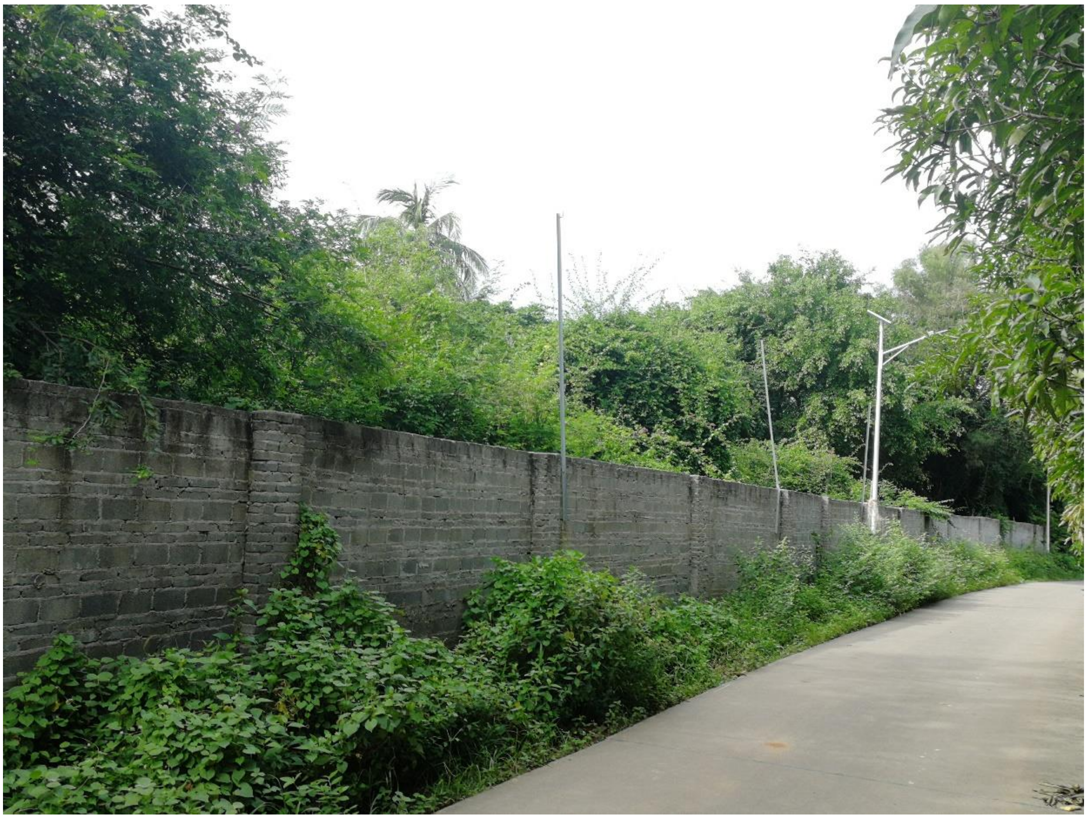

Figure S3. The wall between the monkey park and mango groves

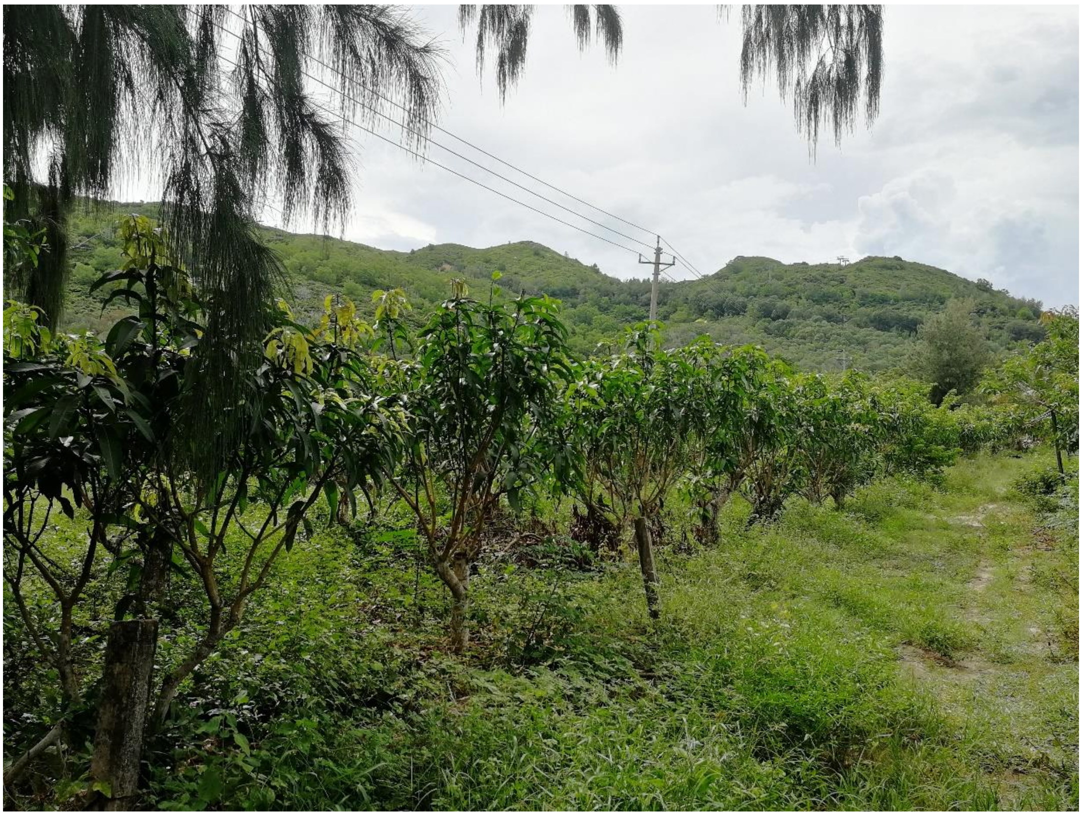

Figure S4. The abandoned mango groves beside the monkey park and reserve hills

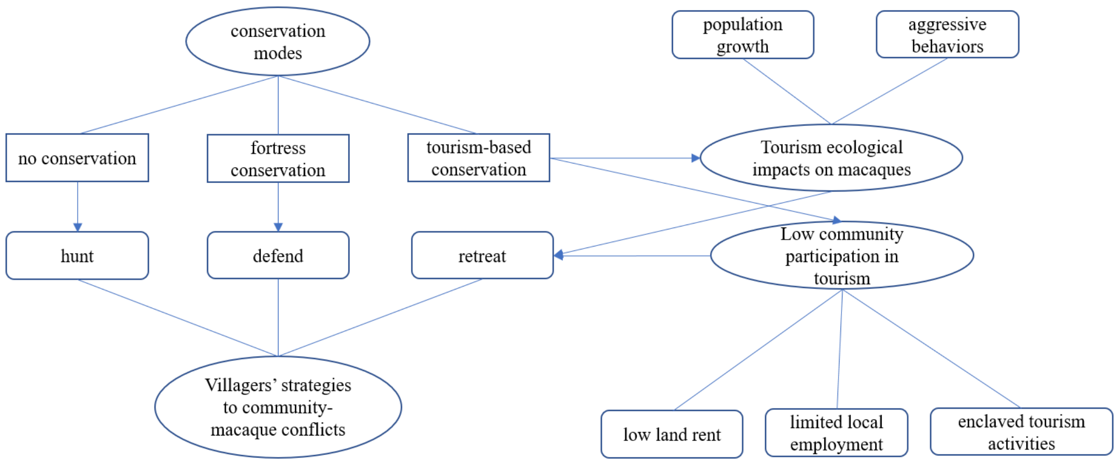

Figure S5. The thematic map showing the (sub)themes from thematic analysis
